# Case report: A rare homozygous variation in the *ENPP1* gene, presenting with generalized arterial calcification of infancy in a Chinese infant

**DOI:** 10.3389/fcvm.2023.1105381

**Published:** 2023-03-03

**Authors:** Pengtao Lu, Jinglong Chen, Mei Chen, Ling Wang, Dandan Xiang, Jie Yin, Shiwei Yang

**Affiliations:** Department of Cardiology, Children's Hospital of Nanjing Medical University, Nanjing, China

**Keywords:** generalized arterial calcification of infancy, ENPP1, early-onset of hypertension, arterial calcification, ectopic mineralization

## Abstract

Generalized arterial calcification of infancy (GACI) is a rare genetic disease characterized by arterial calcifications or stenoses and hypertension. GACI is caused by mutations in the *ENPP1* or *ABCC6* genes, and it often causes intrauterine or early infancy death. Here, we report a case of rare GACI caused by a homozygous variation in *ENPP1*, in a Chinese infant initially presenting with hypertension. The proband was an 8-month-old boy with *in utero* tricuspid valve calcification, presenting with hypertension at birth. Enhanced computed tomography revealed extensive arterial calcification. Genetic testing identified a homozygous variation in *ENPP1* (c.783C > G *p*.Y261X), which led to the diagnosis of GACI. This mutation has been reported in only three Chinese patients, which all initially presented with hypophosphatemic rickets rather than GACI. This case enriches the clinical and genetic spectrum of *ENPP1* mutations and reminds us that GACI should be considered in an infant presenting with hypertension and extensive arterial calcification, and that genetic testing should be performed.

## Introduction

Generalized arterial calcification of infancy (GACI, OMIM 208,000), is a rare autosomal recessive disorder caused by mutations in the *ENPP1* (ectonucleotide pyrophosphatase 1) or *ABCC6* (ATP-binding cassette subfamily C member 6) genes (1,2). GACI is characterized by the deposition of calcium hydroxyapatite in the arteries, skin, and eyes, leading to severe arterial calcification and hypertension; GACI may cause intrauterine or early infant death. GACI is usually treated using bisphosphonates; however, their efficacy is still uncertain. Herein, we report a case of rare GACI in a Chinese infant who initially presented with hypertension and arterial calcification caused by homozygous variation in the *ENPP1* gene.

## Clinical presentation

**Figure 1:**
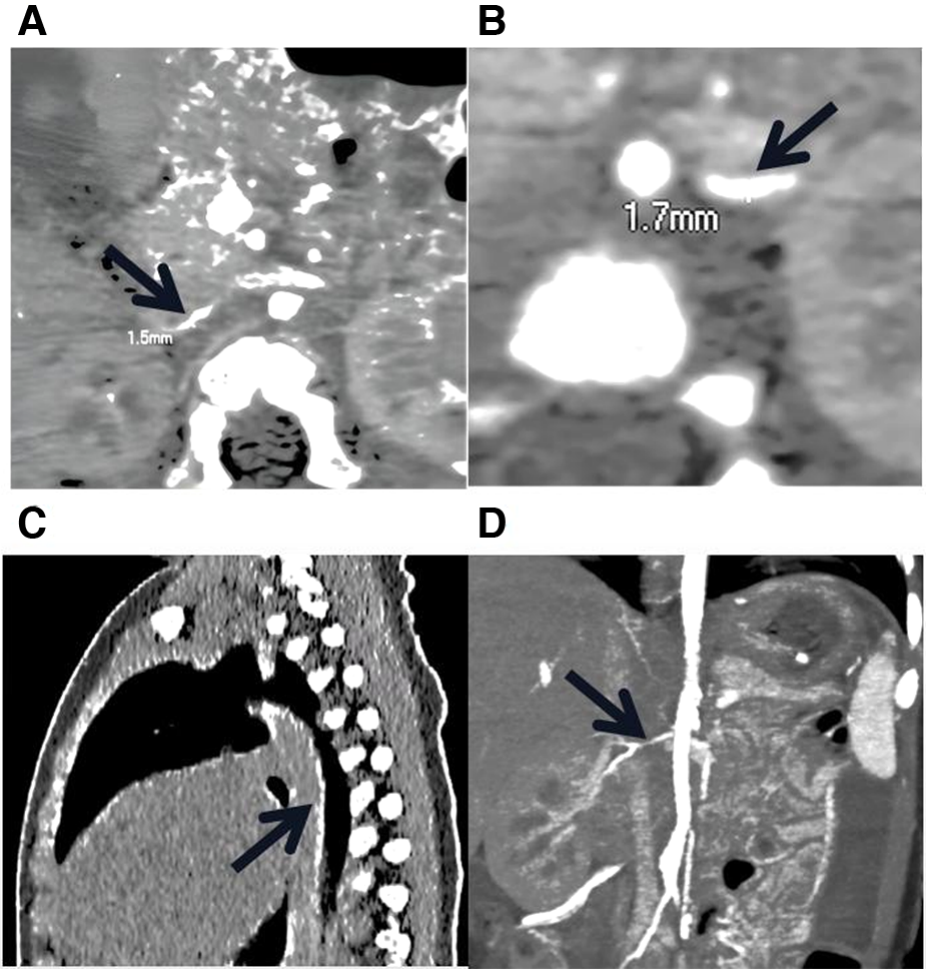
Low-dose, enhanced CT of the child with GACI caused by a homozygous variation in *ENPP1*. (**A**) and (**B**) stenosis of bilateral iliac artery; the thinnest parts of the left and right iliac arteries are 1.7 mm and 1.3 mm wide, respectively. (**C**) The black arrow indicates the presence of calcification in the descending aorta, thoracic aorta, and abdominal aorta. (**D**) The black arrow indicates the stenotic right renal artery.

The proband was an 8-month-old boy admitted to our hospital for hypertension, hypertrophic cardiomyopathy (HCM), and heart failure. Physical examination revealed abnormal vital signs: a heart rate of 180 BPM, a respiratory rate of 46 breaths/min, and a blood pressure of 180/105 mmHg. An electrocardiogram showed sinus tachycardia and left ventricular hypertrophy. Laboratory examinations revealed hypophosphatemia (serum phosphate level of 0.94 mmol/L, normal range 1.5–2.3 mmol/L) and hypocalcemia (serum calcium level of 1.05 mmol/L, normal range 2.25–2.75 mmol/L). Other laboratory results for hereditary and metabolic disorders were negative. Echocardiography revealed hypertrophy of the ventricular wall, with a left ventricular ejection fraction of 62%. Doppler ultrasound revealed bilateral extensive calcification of the renal arteries. Enhanced CT further revealed extensive calcification of the iliac arteries, renal arteries, and abdominal aorta, without bone abnormalities (Figure 1).

The patient is the second son of healthy non-consanguineous Chinese parents. Prenatal concerns discovered on ultrasound included tricuspid valve calcification, fetal HCM, and hydrops fetalis. No abnormality was found in other prenatal investigations. This patient was delivered by emergency caesarean section at 32 weeks of gestation because of an abnormal fetal heart rate. At birth, he presented with neonatal asphyxia, intracranial hemorrhage, shortness of breath, and cyanosis, for which he received ventilator management. The patient developed severe hypertension in the first few hours of life, and echocardiography showed myocardial hypertrophy with no other cardiac structural abnormality. After symptomatic treatment, the patient's condition improved, but the hypertension persisted, and he was discharged from the local hospital on captopril.

During the follow-up, the patient still had hypertension and his condition had deteriorated. He was admitted to our hospital at the age of 8 months due to severe pneumonia and sepsis, and was placed on ventilator and treated with antibiotics and blood pressure reduction. However, his condition worsened, and the hypertension was resistant to triple therapy (nicardipine, metoprolol, and captopril). Eventually, the child died of heart failure and respiratory failure a week after admission, before bisphosphonates could be used. GACI was suspected, and whole exome sequencing was subsequently performed after obtaining the consent of the child's parents. Genomic DNA was extracted from the peripheral blood of the patient and his parents. Whole-exome sequencing was performed in the proband using IDT (Integrated Device Technology, United States) and the x-Gen Exome Research Panel v1.0 whole-exome capture cores. Variants were annotated using the Genome Aggregation Database (gnomAD), 1,000 Genomes Project (Chinese), dbSNP, and the ExAC database. The candidate variants were further validated by Sanger sequencing in the proband and his parents, and the pathogenicity of variants was evaluated according to the American College of Medical Genetics and Genomics (ACMG) criteria. The patient's grandfather, grandmother, and brother were all healthy and refused to accept genetic testing.

**Figure 2:**
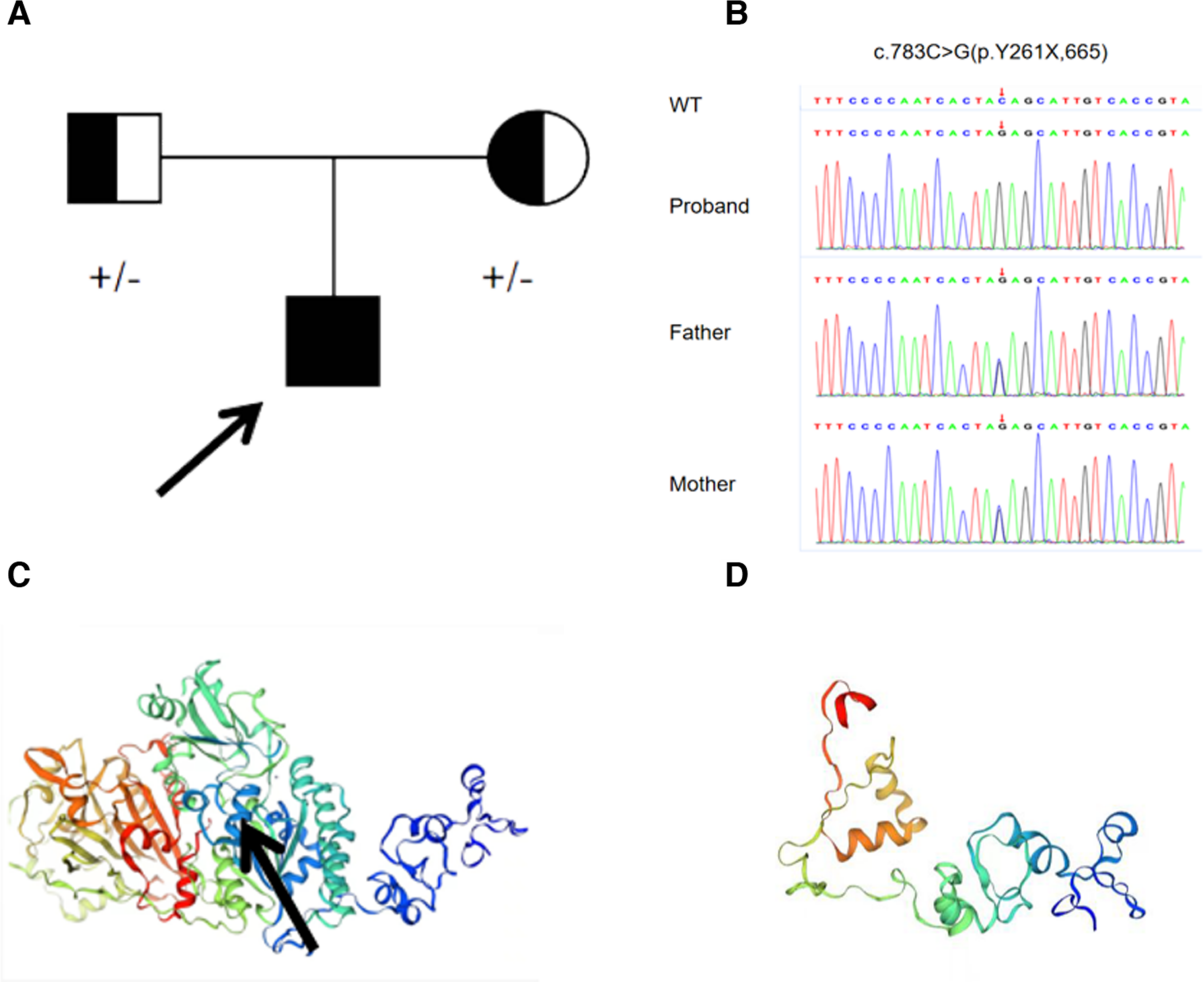
(**A**) family history of the proband. (**B**) Sanger sequencing revealed a homozygous mutation (c.783C > G *p*.Y261X) of *ENPP1* in the proband boy, originating from his parents. (**C**) Normal protein structure; the black arrow represents the mutation site. (**D**) Structure of the mutated protein.

Genetic testing revealed a rare homozygous mutation in the *ENPP1* (c.783C > G Y261X) gene, which was inherited from his parents (Figure 2A, B). This mutation was located in exon 7 of *ENPP1*, and Provean and Mutation-based bioinformatics analysis suggested that this variant was likely a pathogenic mutation. No mutation was identified in the *ABCC6* gene. Based on the clinical and genetic characteristics, this patient was finally diagnosed with GACI.

## Discussion

GACI is a rare but life-threatening disease, secondary to *ENPP1* (75%) or *ABCC6* (9%–10%) mutations (3). To date, only approximately 250 cases of GACI have been reported, with vascular calcification being the earliest and most prominent feature, which is often considered the most significant factor in morbidity and mortality (4). GACI can occur prenatally and has a mortality of approximately 55%; however, many children who died *in utero* and shortly after birth have not been included in this figure, giving an overestimation of the survival rate (5).

Inorganic pyrophosphate (PPi) is a potent calcification inhibitor that acts as a physiological “water softener” by preventing the formation and growth of hydroxyapatite crystals, and *ENPP1* forms adenosine monophosphate and PPi by hydrolyzing extracellular adenosine triphosphate (6). Lack of *ENPP1* results in decreased extracellular PPi, which is linked to ectopic calcification, particularly in the elastic layer of the endovascular lining, cartilage, and other soft tissues. A decrease in plasma PPi levels to almost zero has been suggested to be a potential cause of vascular calcification in GACI (7). A retrospective study by Ferreira et al. found a possible genotype-phenotypic correlation in 55 patients with GACI in a multicenter genetics study: *p*.Pro305Thr in *ENPP1* was related to infant death in five cases, but there was no significant genotype-phenotypic correlation in the other subjects (8). Nitschke et al. found no variants of *ENPP1* or *ABCC6* in 22 patients in a retrospective analysis of 92 patients, implying that other genes may be involved in the onset of the disease.

Chong et al. reported 161 patients with GACI, of which 48% developed the condition *in utero* or in the first few hours of life, namely, “early onset,” and 52% developed the condition after birth (median 3 months), namely, “late-onset” (9). The most common presentations of “early-onset” GACI included fetal hydrops, polyhydramnios, and fetal distress, while “late-onset” GACI was usually asymptomatic *in utero*, but presented with cardiovascular disease, respiratory distress, hypertension, and feeding difficulties after birth. Early-onset GACI commonly affects the hepatic arteries (81%), aorta (80%), and pulmonary arteries (67%), whereas late-onset GACI predominantly affects the coronary arteries (88%), renal arteries (55%), and pulmonary arteries (49%). Nitschke et al. (10) retrospectively analyzed 92 cases of GACI that had been tested for *ENPP1* and *ABCC6* and found that calcification may involve extravascular sites, including the myocardium, pancreas, liver, and kidneys, resulting in multi-organ dysfunction. However, the aforementioned studies were conducted in multiple centers without standardized data collection, limiting the development of consistent management strategies for GACI due to critical knowledge gaps.

The most reported therapy is bisphosphonates, whose efficacy has not been fully confirmed and for which clinical trials are not feasible. *ENPP1* substitution is a potential therapy. *ENPP1*-Fc replacement can prevent vascular calcification and reduce mortality in mice with *ENPP1* variants (11), as well as lower the blood pressure and improve heart function. Against this backdrop, prospective and longitudinal studies on the natural history of GACI are urgently needed to treat arterial calcification to prevent early mortality.

Our patient, presenting with *in utero* hydrops fetalis, tricuspid valve calcification, neonatal asphyxia, intracranial hemorrhage at birth, refractory hypertension, HCM after birth, and extensive calcifications of the bilateral iliac arteries, renal arteries, and abdominal aorta, conformed to “early-onset” GACI. Genetic testing revealed a homozygous variation in *ENPP1* (c.783C > G). This variation results in the deletion of the *ENPP1* protein from the 261 amino acid, Y261X, in the pathogenic form of PVS1, according to ACMG (Figures 2C,D). The mutation has been reported in only three Chinese patients, one with a homozygous variation in *ENPP1* (c.783C > G) and the other two with compound heterozygous mutations (12). Interestingly, the clinical phenotype of all three patients was hypophosphatemic rickets, a skeletal mineralization disorder characterized by excessive renal excretion of phosphorus, resulting in hypophosphatemia, rather than GACI. This phenomenon was also found in the study of Rutsch et al., in which the proband had GACI caused by a homozygous variation in c.2320C > T; however, his father, who had the same homozygous variation, grew up with HR, not GACI (13). This suggests the diversity of the clinical phenotypes in *ENPP1* mutations.

In conclusion, GACI has early-onset symptoms and a very poor prognosis, and early diagnosis of GACI is very important. This report enriches the clinical and genetic spectrum of GACI. Clinicians should suspect GACI when children develop hypertension or vascular calcification soon after birth (median time 3 months). Imaging tests, such as magnetic resonance imaging and computed tomography, as well as genetic testing should be performed.

## Data Availability

The original contributions presented in the study are included in the article/Supplementary Material, further inquiries can be directed to the corresponding author.
